# Green synthesis of silver nanoparticles using *Achillea arabica* and their antioxidant, anti-inflammatory, antidiabetic, and antimicrobial activities

**DOI:** 10.1186/s11671-026-04586-7

**Published:** 2026-05-11

**Authors:** Mohammad A. Alrofaidi, Amani Naouar, Nasser A. A. Ali, Adel Alghamdi, Badiaa Essghaier, Jawher Abdelhak

**Affiliations:** 1 Department of Pharmaceutical Chemistry, Faculty of Pharmacy, Al-Baha University, P.O. Box 1988, Al-Baha, Saudi Arabia; 2 Department of Pharmacognosy and Herbal Medicine, Faculty of Pharmacy, Al-Baha University, P.O. Box 1988, Al-Baha, Saudi Arabia; 3 Department of Pharmacology and Toxicology, Faculty of Pharmacy, Al-Baha University, P.O. Box 1988, Al-Baha, Saudi Arabia; 4 Biochemistry and Biotechnology Laboratory LR01ES05, Faculty of Sciences of Tunis, University Tunis Elmanar, Tunis, Tunisia; 5 Laboratory of Materials, Crystallochemistry and Applied Thermodynamics LR15ES01, Department of Chemistry, Faculty of Sciences of Tunis, University Tunis Elmanar, Tunis, Tunisia

**Keywords:** *Achillea arabica*, Silver nanoparticles, Green synthesis, Antioxidant, Antimicrobial, Anti-inflammatory, Antidiabetic

## Abstract

Green synthesis of metallic nanoparticles using plant extracts has emerged as an environmentally friendly and sustainable alternative to conventional chemical and physical fabrication methods. In the present study, silver nanoparticles (AgNPs) were biosynthesized using aqueous extracts of *Achillea arabica* leaves and flowers, which served as natural reducing and stabilizing agents. The formation of nanoparticles was initially confirmed by a rapid color change of the reaction mixture and further characterized using UV–visible spectroscopy, Fourier-transform infrared spectroscopy (FTIR), X-ray diffraction (XRD), scanning electron microscopy (SEM), and energy-dispersive X-ray spectroscopy (EDX). UV–Vis spectra revealed characteristic surface plasmon resonance peaks at 450 nm for leaf-derived nanoparticles (AgNPsLe) and 490 nm for flower-derived nanoparticles (AgNPsFl), confirming successful nanoparticle formation. XRD analysis indicated the crystalline nature of the nanoparticles with a face-centered cubic (fcc) silver structure, with crystallite sizes ranging from 13–20 nm. SEM micrographs revealed distinct morphological differences, where AgNPsLe predominantly exhibited cubic structures, while AgNPsFl were mainly spherical, suggesting that phytochemical composition influences nanoparticle nucleation and growth orientation. The biological activities of the extracts and synthesized nanoparticles were assessed through antioxidant, antibacterial, and antibiofilm assays. The flower extract showed the highest 1,1-diphenyl-2-picrylhydrazyl (DPPH) radical scavenging activity (81.68%), whereas AgNPsLe demonstrated the strongest Ferric Reducing Antioxidant Power (FRAP) activity with a Half maximal inhibitory concentration (IC_50_) of 0.139 mg mL^− 1^. Antibacterial evaluation revealed that AgNPsFl exhibited the highest antimicrobial potency with Minimum Inhibitory Concentration (MIC) values ranging from 37.5–75 μg mL^− 1^, while AgNPsLe showed MIC values between 75 and 150 μg mL⁻^1^. Furthermore, the synthesized nanoparticles significantly inhibited bacterial biofilm formation with inhibition rates reaching approximately 64%.These findings highlight the potential of *A. arabica*-mediated AgNPs as promising bioactive nanomaterials for antimicrobial and biomedical applications, emphasizing the role of plant-derived phytochemicals in controlling nanoparticle morphology and biological activity.

## Introduction

Nanotechnology is a rapidly emerging area due to its utilization in technology and science for the construction of novel materials at the nanoscale. Nanoparticles (NPs) are described as particles with at least one exterior dimension between 1 and 100 nm, categorizing them within the nanoscale range [69]. Currently, NPs are utilized in different fields, including medicine, agriculture, food industry, healthcare, water treatment, and cosmetics, which improve the quality of products [40]. Additionally, nanoparticles can be exploited in pharmacy to augment the effectiveness of medicinal plant extracts and plant-derived active compounds, alter their release mechanisms, and minimize their adverse side effects [38].

In recent years, “green nanotechnology” has gained increasing attention as it aims to reduce the use of hazardous solvents and toxic reducing agents, while providing safer and more sustainable routes for nanoparticle preparation [22, 35]. Plant-extract-mediated synthesis has emerged as a particularly attractive strategy because reaction parameters such as pH, temperature, reaction time, and precursor concentration can be adjusted to tune nanoparticle yield, stability, and size distribution, which are critical determinants of biological performance [22]. At the same time, recent reviews also emphasize that reproducibility, batch-to-batch consistency, and safety/biocompatibility should be considered when proposing biomedical or environmental applications of AgNPs [35].

Silver nanoparticles (AgNPs) are particularly attractive among inorganic metallic nanomaterials, because they combine powerful antimicrobial effects, strong plasmonic optical properties, high conductivity, catalytic activity and relatively low cost, making them suitable for applications in medicine, electronics, sensing, and environmental technologies, owing to their distinctive physicochemical properties and broad-spectrum biological activities [36, 65].

Silver's therapeutic and preventive activity has been documented for centuries [61], and more recently, AgNPs have demonstrated various biological activities such as antibacterial, anticancer, antioxidant, antidiabetic, and antiviral activity [19, 60], Essghaier et al. 2024). Notably, biogenic/green AgNPs are increasingly considered a promising approach for bacterial treatment because they act via multi-target antibacterial mechanisms rather than a single site of action. For example, AgNPs can bind to bacterial cell membranes through interactions with sulfur–phosphorous-containing components, disrupt permeability and respiration, inactivate enzymes and proteins, and ultimately induce DNA damage resulting in bacterial cell death [54]. Such multi-target actions are particularly relevant in the era of multidrug resistance and support the continued development of green-synthesized AgNPs as alternative antibacterial platforms [54], Sivakami et al. 2025). Accordingly, the present work focuses on in vitro antibacterial assessment, and conclusions are limited to the experimental conditions investigated**.** Among green synthesis techniques for producing nanometals (NMs), plant-mediated synthesis is regarded as one of the most effective due to various advantages, including environmental safety, simplicity, cost-effectiveness, scalability, and the availability of natural reducing and stabilizing agents such as phenolics, terpenes, and polysaccharides. These properties render this approach more advantageous than other biological methods. Rafique et al. 2016; [3, 16]. In this context, recent studies highlight that plant-derived functional groups—such as phenolics and carbonyl-containing constituents—can mediate Ag^+^ reduction and concurrently cap/stabilize the resulting AgNPs, thereby modulating their physicochemical characteristics and, consequently, their biological activity [54, 72, 73].

In addition to antimicrobial applications, recent studies focus on the extensive uses of AgNPs, including their incorporation into polymers and electrospun nanofibers for beneficial coatings and biomedical materials [1], as well as their utilization in sensors, diagnostics, drug delivery formulations, and medical device coatings [48]. Polymers containing AgNP are increasingly investigated for food-packaging applications to improve antibacterial and oxidative stability and lengthen shelf life, but controlled distribution and safety concerns are crucial [57]. In environmental and industrial contexts, green-synthesized AgNPs have been examined for their applications in water treatment and catalytic processes, where surface chemistry and stability significantly impact functionality efficacy [22]. In pharmacy and medicine, AgNPs are extensively studied as antimicrobial agents in topical formulations and biomedical materials, such as wound dressings, hydrogels/nanogels, and medical-device coatings, where regulated Ag^+^ release and surface interactions can inhibit pathogenic proliferation and biofilm development [2, 48, 63]. Recent studies and reviews focused on formulations highlight the value of AgNP-loaded hydrogels as antibacterial wound dressings, demonstrating efficacy against drug-resistant bacteria, while emphasizing the necessity of combining efficacy with biocompatibility [2, 33].

Concerning metabolic disorders, silver nanoparticles (AgNPs) have been investigated for antidiabetic purposes, specifically through in vitro inhibition of carbohydrate-hydrolyzing enzymes (α-amylase and α-glucosidase). Recent studies have demonstrated glucose-lowering effects in animal models utilizing green-synthesized AgNPs, requiring a further studies of nano-enabled methods to treat metabolic disorders [25, 27, 28].

Moreover, recent in vivo studies have shown the anti-inflammatory activity of AgNPs, indicating modulation of pro-inflammatory mediators and COX-2-related pathways; however, these effects are dependent on particle characteristics and dosing, highlighting the necessity for comprehensive safety and biocompatibility assessments [11].

Emerging concerns over the side effects, resistance, and ecological consequences of synthetic drugs have accelerated the search for natural compounds possessing anti-inflammatory, antidiabetic, and antimicrobial effects [66] with fewer side effects. Medicinal plants provide a substantial source of bioactive compounds, including flavonoids, alkaloids, terpenoids, and phenolics, which exhibit therapeutic efficacy and function as efficient reducing and capping agents in the eco-friendly synthesis of nanoparticles [12, 58, 68]. For instance, silver nanoparticles (AgNPs) synthesized using *Azadirachta indica* (neem) extract have shown marked antimicrobial and wound-healing properties, and other phytofabricated AgNPs (e.g., from *Anisomeles ovata* flower buds) have also demonstrated in vitro antimicrobial activity; in contrast, curcumin nanoparticles (turmeric) display promising antidiabetic and anti-inflammatory effects [9, 44, 53, 59]. Similarly, multiple recent green-nanotechnology studies report dose-dependent antimicrobial effects of plant/fungal-assisted nanoparticles, often accompanied by anti-inflammatory activity, highlighting their potential as multifunctional therapeutic platforms [71–73]. The *Achillea* genus comprises over 120 species and exhibits a wide range of biological activities, including antioxidant, anti-inflammatory, analgesic, antipyretic, antidiabetic, antibacterial, anthelmintic, and antihypertensive properties. These species have been used traditionally to treat wounds, bleeding, headaches, inflammation, pains, spasmodic diseases, flatulence, and dyspepsia [70]. Sesquiterpene lactones, flavonoids, triterpenes, sterols, coumarins, and phenolic acids were reported in the genus [8].

*A. arabica* Kotschy is commonly referred to as “Althufra” in Saudi Arabia and employed in folkloric medicine as a decoction for treating several ailments, namely abdominal pain, wound healing, jaundice, and other hepatic disorders. Literature reports have revealed different biological activities of *A*. *arabica*, namely antidiabetic, antioxidant, antibacterial, antifungal, and hepatoprotective activity [4, 32].

Previously, limited studies were performed on the biosynthesis of AgNPs from medicinal *Achillea* species, namely *A. biebersteinii* [6]. *A. wilhelmsii* C. Koch (Aw) [13], *A. millefolium* [37], *A. fragrantissima* [5], and *A. maritima* [20].

To date, there is no study performed regarding the synthesis of AgNPs mediated by *A. arabica* leaf and flower extracts for evaluating their anti-inflammatory, antidiabetic, antimicrobial and antioxidant activities. Therefore, the present work aims to green-synthesize AgNPs using leaf and flower extracts of *A. arabica*, to characterize the resulting nanoparticles using standard physicochemical techniques, and to evaluate their antioxidant, anti-inflammatory, antidiabetic, and antimicrobial activities under in vitro conditions employed. Nevertheless, further studies are required to clarify safety/cytocompatibility and to validate efficacy in vivo before any clinical application.

## Materials and methods

### Plant sampling and preparation of plant extract

**Fig. 1:**
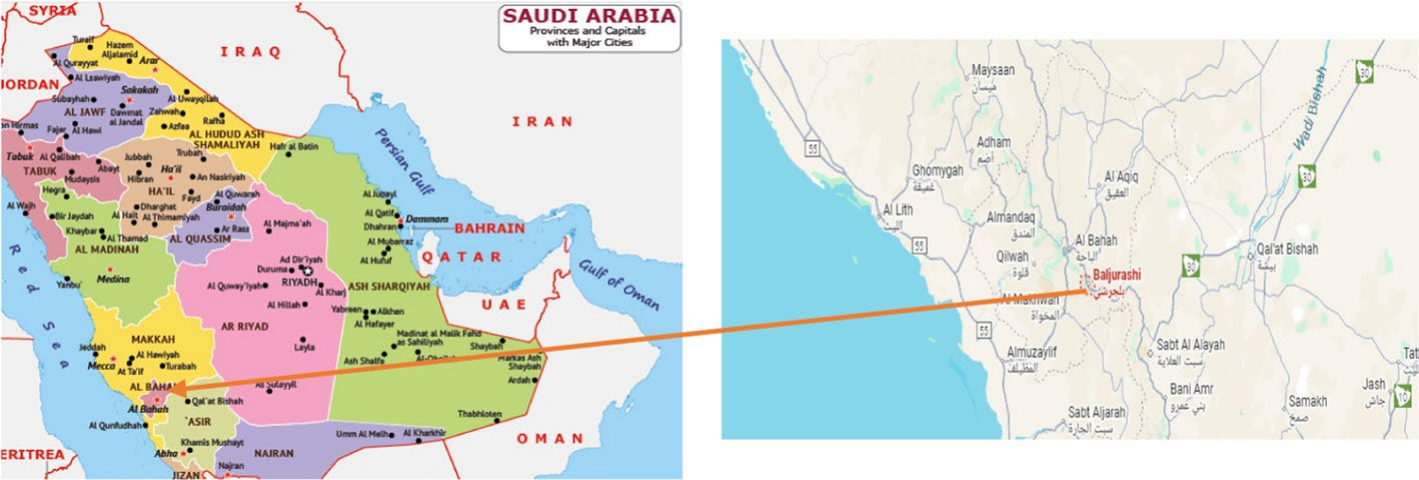
Map of Saudi Arabia showing the location of the sample xerophyte cottonweed species *A. arabica* used for the green synthesis of silver nanoparticles

*A. arabica* was wild-collected from Baljurashi district (Fig. 1), Al-Baha region, Saudi Arabia, during March–April 2024 (Approximate coordinates: 19° 52′ 31.0″ N 41° 33′ 10.7″ E). Taxonomic identification was performed by botanist Dr. Othman S. S. Al-Hawshabi, and a voucher specimen (CP‑101) belonging to the family Asteraceae was deposited in the Pharmacognosy Department, Faculty of Pharmacy, Al Baha University, Saudi Arabia. The plant material was collected from non-protected area, and no specific permits were required according to local regulations. Furthermore, no endangered or protected species were involved in this study.

Twenty grams of flower and leaf powders were weighed into a beaker and separately added to 100 mL of distilled water. The mixtures were maintained at 37 °C for 24 h. The extracts were filtered through filter paper and stored in the dark at 4 °C until use for the green synthesis of silver nanoparticles.

### Green synthesis of silver nanoparticles

The green synthesis of AgNPs using aqueous extracts of *A. arabica* flowers and leaves, was performed according to the method described by [20].

Briefly, an aqueous silver nitrate (AgNO_3_) solution (5 mM) was freshly prepared by dissolving analytical-grade AgNO_3_ in distilled water. For the synthesis of AgNPs, 5 mL of each plant extract was mixed with 20 mL of AgNO_3_ solution (5 mM) in a 100 mL Erlenmeyer flask. The reaction mixture was stirred at room temperature (25 ± 2 °C) for 5 min and then incubated in the dark for 24 h. The formation of AgNPs was indicated by a color change of the reaction mixture from pale yellow to dark brown due to surface plasmon resonance. The synthesized nanoparticles were subsequently recovered by centrifugation at 10,000 rpm for 15 min, washed three times with distilled water to remove residual biomolecules, and finally dried at 40 °C for further characterization and biological assays [21].

### Characterization of silver nanoparticles

The characterization was done by UV-Vis spectroscopy, X-ray diffraction (XRD), and Fourier transform infrared spectroscopy (FTIR). The reduction of silver ions was confirmed via UV-Vis spectroscopy using distilled water as a blank with measurements performed on a UNICO 20802 spectrophotometer over 250–700 nm, as previously described by [19, 76], the chosen wavelength range covers most UV and visible absorptions for organic/inorganic analytes, the scan speed is 1–2 nm per min, the baseline is zeroed with blank and the temperature is the room temperature (between 20 and 25 °C). FTIR analysis spectra were recorded from 4000 to 400 cm^− 1^ on a Varian FTIR 640 using KBr pellets, spectra were collected at a resolution of 4 cm^− 1^ with 32 scans at room temperature. XRD analysis was conducted on a D8 ADVANCE BRUKER diffractometer with Cu-Kα radiation (λ = 1.5406 Å) and a Lynxeye detector [26, 46], the diffraction patterns were recorded over a 2θ range of 10–80° and a scanning rate of 0.5–2° min^− 1^ at 40 kV and 40 mA. A Scanning electron microscope SEM was used to examine the morphology, composition and structure of the biosynthesized silver nanoparticles derived from the flowers AgNPsFl and leaves AgNPsLe extracts of *Achillea arabica* using a TS Quanta 250. The SEM measurements were carried out under the following typical conditions: the accelerating voltage: 5–30 kV, the magnification range: ~ 30 × to 300,000 × , the working distance: ~ 7–10 mm and the resolution: ~ 1–1.2 nm at 30 kV in high-vacuum mode.

### Biological activities of plant extracts and the silver nanoparticles

#### Antioxidant activity determination

##### **Radical scavenging activity on (2,2-diphenyl-1-picrylhydrazyl) (DPPH) radical**

The radical scavenging activity of the extracts was evaluated using the 2,2-diphenyl-1-picrylhydrazyl (DPPH) assay as described [19]. Briefly, 20 μL of each sample solution was mixed with 200 μL DPPH methanolic solution (120 μM prepared in 96% methanol). The reaction mixture was incubated in the dark at room temperature for 30 min. The absorbance was then measured at 517 nm using a microplate spectrophotometer. The radical scavenging activity was expressed as percentage inhibition (%) relative to the negative control. Butylated hydroxytoluene (BHT) was used as a positive control at a concentration of 1 mg/mL.

##### **Ferric reducing antioxidant power (FRAP)**

The ferric reducing antioxidant power of the samples was determined following the method described by [64]. Briefly, 50 μL of sample solution was mixed with 50 μL distilled water and 50 μL of 1% potassium ferricyanide [K_3_Fe(CN)_6_]. The mixture was incubated at 50 °C for 20 min. Subsequently, 50 μL 10% trichloroacetic acid (TCA) and 10 μL of 0.1% ferric chloride (FeCl_3_) were added and the mixture was further incubated for 10 min at 50 °C. The absorbance was measured at 700 nm using a Multiskan Microplate Spectrophotometer. In this assay, Increased absorbance indicates higher reducing power of the tested samples reflecting the ability of the sample to convert Fe^3+^ to Fe^2+^. BHT was used as a reference antioxidant.

#### Total antioxidant capacity

This assay is based on the reduction of Mo(VI) to Mo(V) by tested samples and the subsequent formation of a green phosphate/Mo(V) complex at acid pH [19, 31]. An aliquot of 0.1 mL of each sample was combined to 1 mL of reagent solution (0.6 M sulfuric acid, 28 mM sodium phosphate and 4 mM ammonium molybdate). The tubes were incubated at 95 °C for 90 min. After that, the mixture was cooled at room temperature and the absorbance of each solution was measured at 695 nm against a blank. The ascorbic acid was used as a standard, and the total antioxidant capacity (TAC) was expressed as mg ascorbic acid equivalent per gram dry weight (mg AAE/g DW).

#### In vitro anti-inflammatory activity

The Anti-inflammatory activity of the aqueous extract from leaves and flowers as well as their respective silver nanoparticles was evaluated based on the method of Fetni et al. [23]. This consists of evaluating their ability to inhibit Bovine serum albumin (BSA) denaturation after incubation of the mixture (the containing BSA and the sample) at 37 °C for 20 min. the absorbance was measured at 660 nm to determine the level of protein denaturation and the percentage of inhibition, I(%) was calculated based on the Eq. (1) as follows:

1 $$I\% = 100 \times \left( {\frac{Ac - As}{{Ac}}} \right)$$

where, as (sample Absorbance): Absorbance of the reaction mixture (BSA 1% + sample) and Ac (Control Absorbance): Absorbance of the control mixture (BSA 1%).

#### In vitro antidiabetic activity

The antidiabetic potential of the biosynthesized silver nanoparticles was evaluated through the inhibition of α-amylase and α-glucosidase activity. The reaction mixture consisted of 500 µL of AgNPs solution at different concentrations and 500 μL of α-amylase enzyme solution (α-amylase at 0.5 mg/mL prepared in phosphate buffer, pH 6.9). After pre-incubation at 25 °C for 10 min, 500 µL of 1% soluble starch solution was added as a substrate and the mixture was further incubated for 10 min. The enzymatic reaction was terminated by adding 1 mL of 3,5-dinitrosalicylic acid (DNS) reagent, followed by heating in a boiling water bath for 5 min. After cooling at room temperature, 5 mL of distilled water was added and the absorbance was measured at 540 nm. Acarbose was used as a positive control. The percentage inhibition of α-amylase activity was calculated according to the method described by [21].

#### Antimicrobial capabilities

##### **Agar well diffusion method**

The antibacterial action of samples was investigated against a list of reference strains belonging to *Escherichia coli* ATCC 35218; *Listeria monocytogenes* ATCC 19115; *Staphylococcus aureus* ATCC 43300; *Staphylococcus epidermidis* CIP 106510; *Salmonella typhimurium* ATCC 14028; *Enterococcus faecalis* (ATCC 29212); *Bacillus cereus* ATCC 14579; *Candida albicans* ATCC 90028; *Candida krusei* ATCC 6258. The antibacterial and anti-Candida activities of samples were investigated by the agar well diffusion method as previously described by [19]. The bacterial suspension was adjusted to 10^7^ CFU/mL and the Candida solution adjusted to 10^6^ spores/mL. The Mueller-Hinton media (Biorad, France) and the Sabouraud chloramphenicol agar were used for antibacterial and antifungal assays, respectively. The culture plates were incubated at 37°C for 24 h. Each test was performed in triplicate. Levofloxacin (5 µg) and amphotericin B (25 µg mL^− 1^) were used as a positive standard.

##### **MIC and MBC determinations**

The minimum inhibitory concentration (MIC) of the synthesized silver nanoparticles was determined using the broth microdilution method in sterile 96-well flat-bottom microplates. The MIC was defined as the lowest concentration of nanoparticles that showed no visible bacterial growth after incubation. To determine the minimum bactericidal concentration (MBC), 10 µL from the wells showing no visible growth (MIC and higher concentrations) were subcultured onto agar plates and incubated for 24 h. The appearance of bacterial colonies indicated a bacteriostatic effect, whereas the absence of colony formation confirmed the bactericidal activity (MBC) of the tested silver nanoparticles [19].

##### **Biofilm inhibition**

The effect of the biosynthesized silver nanoparticles on biofilm formation was evaluated based on the previously described methods by [42] and [29] by using the 96-well flat-bottom microplates.

##### **Silver nanoparticles effect on the bacterial cell wall**

***Lysozyme action***

*Bacillus cereus* and *Staphylococcus aureus* species were grown on agar medium for 24 h at 37 °C, then the cells were washed with distilled water and suspended in phosphate buffer (50 mM, pH = 6.2). In an Eppendorf tube, 100 µl bacterial cell wall suspension was mixed 50 µL of the tested sample. The reaction mixture was incubated at 37 °C for 60 min. Lysozyme activity was expressed as arbitrary unit AU/mL/min and calculated from the decrease in optical density at 600 nm [62]

***Lipopolysaccharide degradation***

The effect of the synthesized silver nanoparticles on lipopolysaccharide (LPS) degradation was evaluated according to the method described by Essghaier et al. [20]. Briefly, Gram-negative bacterial cells were grown in 1 mL of LB broth in the presence or absence of AgNPs (150 μg/mL) at 37 °C for 18 h under shaking conditions (120 rpm). The cultures were centrifuged at 8000 rpm for 10 min and the obtained cell pellets were washed and resuspended in 0.9% saline solution (NaCl). Subsequently, an equal volume of 5% phenol solution was added, followed by five volumes of concentrated sulfuric acid (H_2_SO_4_). The reaction mixture was incubated in the dark for 1h, and the absorbance was measured at 490 nm to estimate LPS degradation.

**Statistical analysis**

Values are presented as mean ± standard error of the mean (SEM). Multiple comparisons were performed using the Student–Newman–Keuls (SNK) test at a significance threshold of 5% (*p* < 0.05). Means sharing the same letter are not significantly different (n = 3).

## Results

### Characterization of silver nanoparticles derived from leaves and flowers of *A. arabica* species

#### UV-visible spectroscopy analysis

**Fig. 2:**
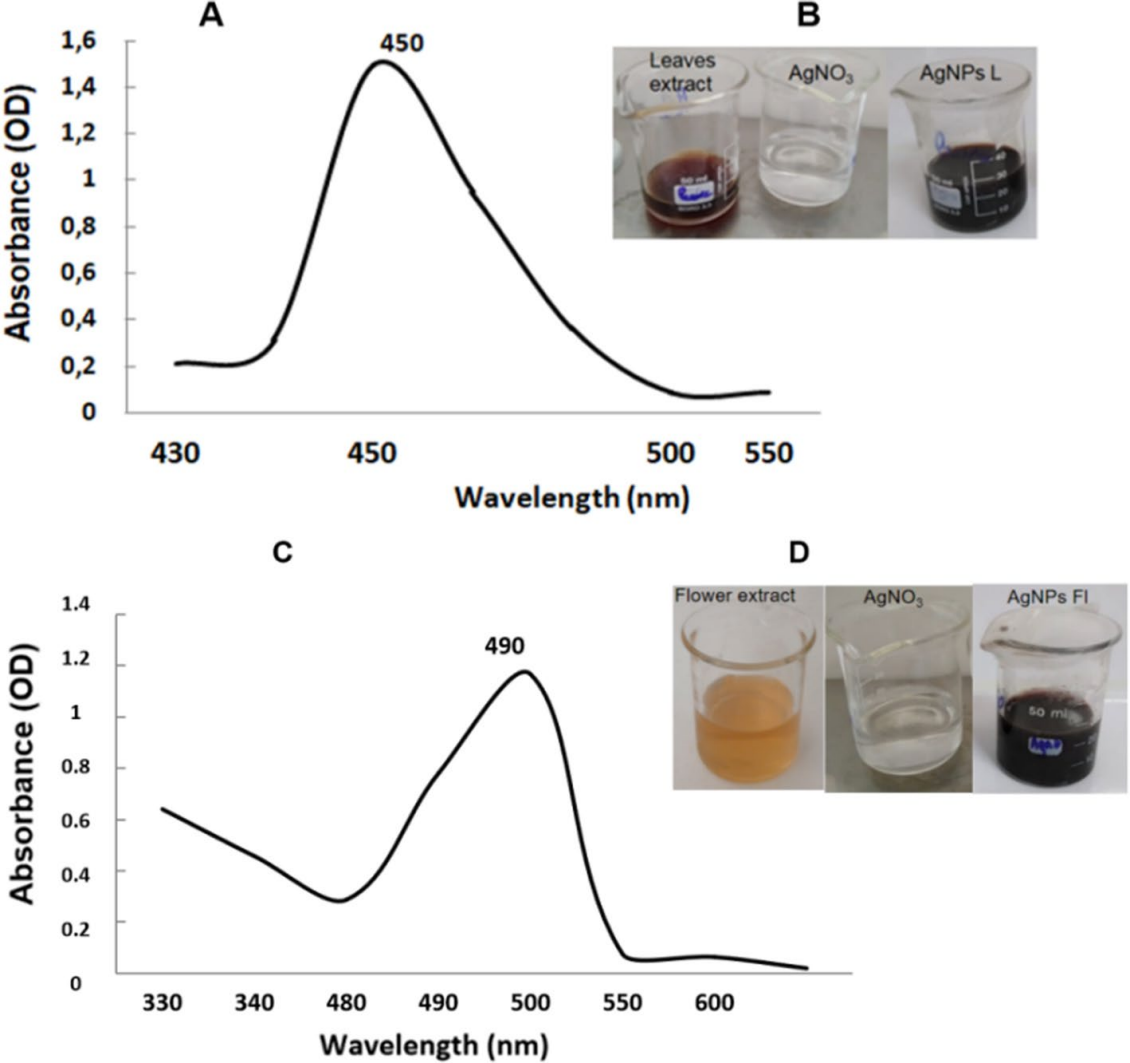
Visual appearance of the leaves extract (**B**), the flowers extract (**D**) and the corresponding synthesized silver nanoparticles, UV-Visible spectrum of green synthesized silver nanoparticles AgNPsLe (**A**) and AgNPsFl (**C**)

The synthesis of AgNPs was evidenced by a visible color change of the reaction mixture, shifting from pale yellow to brown (Fig. 2B, Fig. 2D). This variation strongly suggests the reduction of Ag^+^ ions to metallic silver (Ag^0^).

UV-Vis spectrum further confirmed silver nanoparticle formation, displaying a distinct absorption peak at 450 nm for AgNPs biosynthesized from Leaves extract and 490 nm for AgNPs from flowers extract (Fig. 2A, c).

#### FTIR analysis

**Fig. 3:**
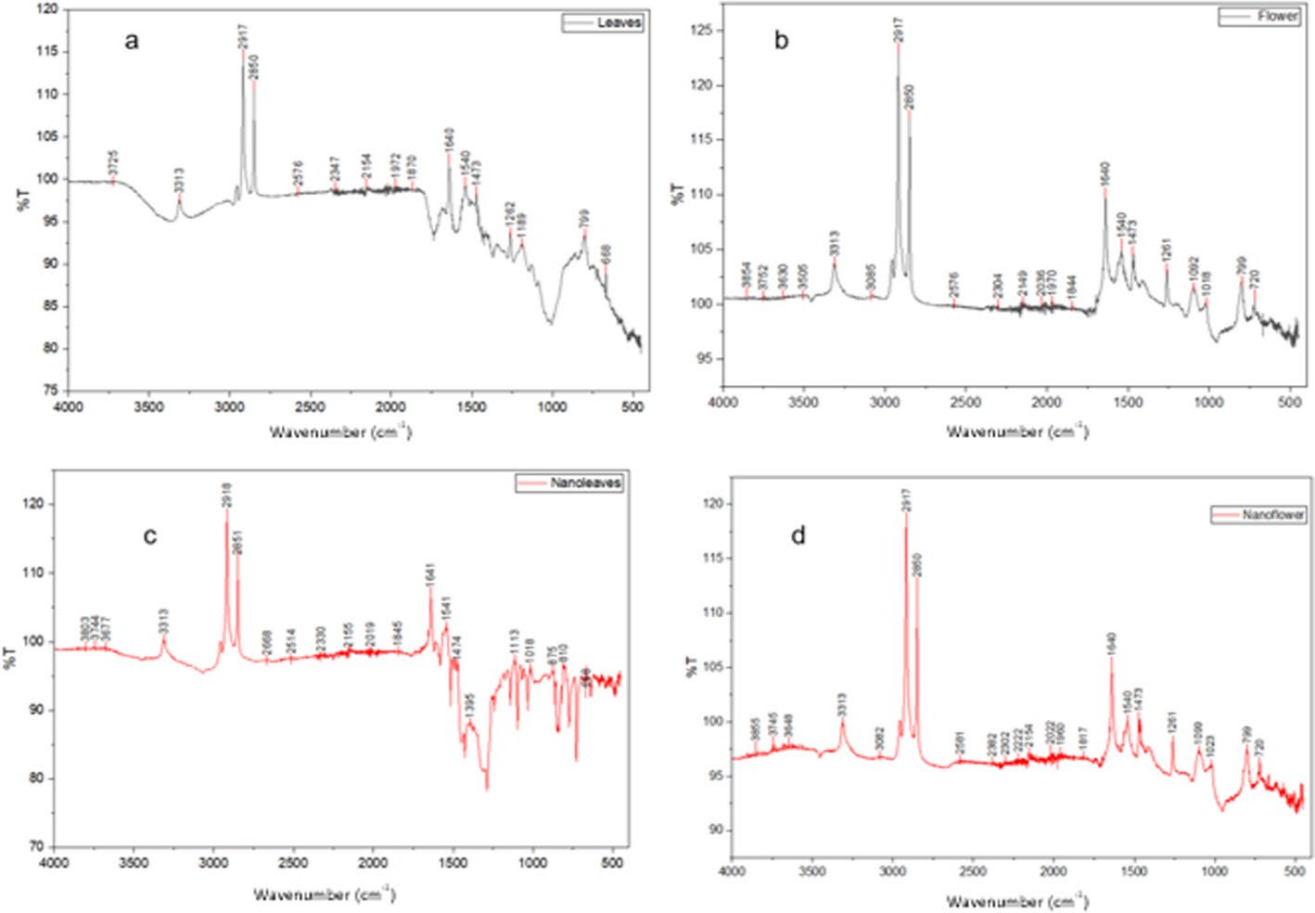
FTIR analysis of leaves (**a**) nanoparticles derived from Leaves extract (**c**), Flowers (**b**) and nanoparticles derived from flowers extract (**d**)

The FTIR spectra (Fig. 3 a–d) provide clear evidence of the functional groups present in the plant extracts (leaves and flowers) and their corresponding synthesized nanoparticles, confirming the successful bioreduction and stabilization processes.

In the spectrum of the leaves extract (Fig. 3a), the broad absorption band observed around 3325 cm^− 1^ corresponds to the stretching vibration of hydroxyl (–OH) groups, indicating the presence of polyphenols, flavonoids, and other alcohol-based compounds. The distinct peaks near 2913 cm^− 1^ and 2850 cm^− 1^ are attributed to C–H stretching vibrations of aliphatic chains. The band at 1640 cm^− 1^ corresponds to C = O stretching vibrations of amide or carboxylic groups, while peaks around 1419–1385 cm^− 1^ are associated with C–N stretching and –CH bending of aromatic amines. Absorptions at lower wavenumbers (around 1010 cm^− 1^ and 669 cm^− 1^) indicate C–O–C and C–H bending modes of polysaccharides and aromatic compounds, respectively [55].

Similarly, the flowers extract spectrum (Fig. 3b) exhibits a strong –OH band at 3313 cm^− 1^ and characteristic C–H stretching peaks at 2917 cm^− 1^ and 2850 cm^− 1^, consistent with the presence of organic biomolecules. The band at 1640 cm^− 1^ confirms carbonyl stretching vibrations, while absorptions between 1450–1380 cm^− 1^ indicate the presence of C–N and C = C aromatic modes. Peaks in the region 1100–600 cm^− 1^ further confirm C–O and C–H bending vibrations, which are typical of phenolic and terpenoid constituents.

Upon nanoparticles synthesis, significant spectral modifications are evident. In the leaves-derived nanoparticles (Fig. 3c), the broadening and slight shift of the –OH band (~ 3313 cm⁻^1^) and the attenuation of C = O and C–N peaks (~ 1641 cm^− 1^ and 1541 cm^− 1^) suggest the active participation of these functional groups in the reduction of metal ions and the stabilization of nanoparticles. The appearance of new or intensified peaks in the fingerprint region (700–1000 cm^− 1^) indicates metal–oxygen bond formation (M–O), confirming successful nanoparticles formation.

Similarly, the flowers-derived nanoparticles (Fig. 3d) maintain the key –OH and C–H bands but exhibit noticeable shifts and reduced intensity in the carbonyl and amine regions, implying coordination of oxygen and nitrogen donor groups with the nanoparticles surface. The persistence of organic functional groups also indicates capping by biomolecules, providing stability and preventing agglomeration.

A comparison before and after nanoparticle formation shows that the flower and leaf extracts exhibit intense O–H, C = O, and amide bands, representing the phytochemical compounds responsible for the reduction.

Meanwhile, the nanoflowers and nanoleaves show slight shifts in the O–H, C = O, and Amide bands. These variations in intensity indicate the binding of biomolecules to the surface of the nanoparticles and the formation of encapsulated nanoparticles. The peak shifts confirm the interaction between metal ions and functional groups.

The proposed green synthesis mechanism involves four steps. The first is reduction, where the phenolic –OH and carbonyl groups donate electrons according to the following reaction (1):

$$M^{n + } + phytochemicals \to M^{0} \left( {nanoparticles} \right)$$

This shows that flavonoids and polyphenols reduce the concentration of metal ions and proteins can facilitate electron transfer. The second stage is nucleation, where we observe the aggregation of reduced metal atoms and the formation of nuclei through small aggregates. The third is growth; controlled growth occurs, and biomolecules regulate particle size.

The final step is the stabilization (capping) of proteins (amide groups), polyphenols (–OH, C = O), and polysaccharides (C–O–C). This involves attaching the nanoparticles to their surface through coordination bonds, electrostatic interactions, and hydrogen bonds. This prevents agglomeration, oxidation, and uncontrolled growth.

FTIR spectra clearly demonstrate that phenols and flavonoids (–OH) act as reducing agents, proteins (amide I and II) act as capping/stabilizing agents, carbonyl groups (C = O) coordinate with the metal surface, while polysaccharides (C–O–C) provide steric stabilization.

The peak shifts observed in nanoflowers and nanoleaves confirm that these biomolecules are directly involved in reduction and stabilization, supporting a green synthesis mechanism where plant phytochemicals act as both reducing and capping agents.

#### XRD spectrum

**Fig. 4:**
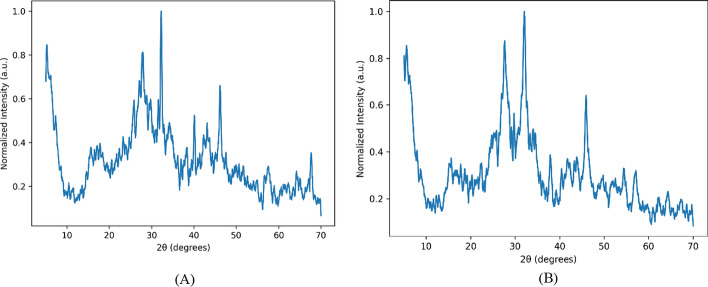
XRD diffraction pattern of silver nanoparticles derived from leaves AgNPsLe (**A**) and derived from flowers AgNPsFI (**B**) of plant species *A. arabica*

The crystalline nature of nanoparticles was determined via X-ray diffraction. For the leaves sample (Fig. 4A), the X-ray diffraction pattern shows several diffraction peaks corresponding to several diffraction peaks. These peaks correspond to the face-centered cubic (fcc) crystalline structure of silver nanoparticles (AgNPs). The main characteristic reflections of metallic silver typically appear near 38° which corresponds to (111) plane, 44° which corresponds to (200) plane, 64° and 77° which can be attributed to (220) plane and (311) plane, respectively. The strong peak around 32–38° suggests that the (111) crystallographic plane is dominant, which is common in biosynthesized silver nanoparticles because this plane has the lowest surface energy and highest stability. However, the pattern also shows a broad background hump between ~ 15° and 35°, which indicates the presence of amorphous organic material. This is typical in green synthesis using plant extracts, where biomolecules from the extract remain adsorbed on the nanoparticle surface. These biomolecules (polyphenols, proteins, flavonoids, polysaccharides) act as reducing agents (Ag^+^ → Ag^0^) and Capping/stabilizing agents, because these compounds are non-crystalline, they generate the amorphous halo observed in the XRD pattern.

X-ray diffraction (XRD) was performed on the AgNPs (Flowers sample, Fig. 4B). The refined X-ray diffraction (XRD) pattern confirms the formation of crystalline silver nanoparticles (AgNPs) synthesized using the plant leaf extract. The main diffraction features correspond to the face-centered cubic (fcc) crystalline structure of metallic silver. The reflections correspond to Bragg diffraction of metallic Ag, confirming the successful reduction of Ag^+^ ions into crystalline Ag^0^ nanoparticles. The (111) plane is usually the dominant peak, indicating that the nanoparticles preferentially grow along this crystallographic direction, which is common in biologically synthesized silver nanoparticles.

The XRD pattern shows a broad diffuse background, which indicates the presence of amorphous organic material. This is expected in green synthesis routes. The amorphous component originates from polyphenols, proteins, flavonoids, terpenoids present in the plant leaf extract. These biomolecules act as reducing agents (convert Ag^+^ → Ag^0^), capping agents (stabilize nanoparticles) and surface functionalizers. Therefore, the amorphous halo does not indicate impurity, but rather the organic coating surrounding the nanoparticles.

Despite the amorphous contribution, the presence of distinct diffraction peaks confirms that the nanoparticles possess a crystalline metallic core.

The size of the nanoparticles was calculated according to Scherrer mathematical equation as follows (Eq. 2):

2 $$d = \frac{k.\lambda }{{\beta cos\theta }}$$

where d is the average crystalline size of the nanoparticles, k is the geometric factor equal to 0.9, $\lambda$ = 1.5406Å, and is the wavelength of X-ray radiation, and $\beta$ is the angular FWHM (full-width at half maximum) of the XRD peak at a diffraction angle of $\theta$ [78]

#### Scanning electron microscopy

**Fig. 5:**
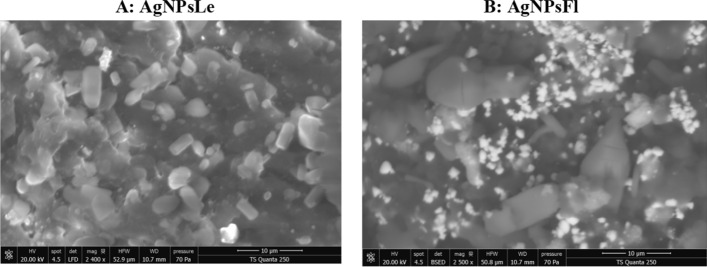
The scanning electron microscopy SEM images of the leaves extract nanoparticles (AgNPsLe) **(A)** and the flowers-derived AgNPs (AgNPsFl) **(B)**

The Scanning Electron Microscopy SEM images obtained using a TES Quanta 250 (Fig. 5) reveal morphological differences between the green synthesized nanoparticles. The leaves-derived AgNPs (AgNPsLe) exhibit predominantly cubic shapes (Fig. 5A), whereas the flowers-derived AgNPs (AgNPsFl) are mainly spherical (Fig. 5B). In addition, some nanoparticles appear larger in the SEM images, which can be attributed to the agglomeration of smaller particles.

According to the provided SEM results, leaves-derived AgNPs (AgNPsLe) show predominantly cubic morphology. Flowers-derived AgNPs (AgNPsFl) are mainly spherical in shape. Some particles appear larger due to agglomeration of smaller nanoparticles. These findings indicate that the plant part used in green synthesis significantly influences nanoparticle morphology.

Previous studies on green synthesized silver nanoparticles commonly report, spherical morphology as the dominant shape, especially when plant extracts are used as reducing and stabilizing agents, or polydispersity in particle size due to variability in phytochemical composition, or agglomeration caused by high surface energy of nanoparticles, and occasional formation of cubic, triangular, or irregular shapes, depending on type of plant extract, concentration of biomolecules and reaction conditions (pH, temperature, and time).

The cubic morphology observed in leaves-derived AgNPs is less commonly reported compared to spherical nanoparticles. This suggests that the phytochemical composition of leaves may promote anisotropic growth, leading to cubic structures. In contrast, the spherical morphology of flowers-derived AgNPs aligns well with most previous green synthesis studies.

The morphological differences may be attributed to variation in phytochemical constituents (flavonoids, phenolics, proteins), different capping and stabilizing mechanisms, differential nucleation and growth rates during nanoparticle formation. Agglomeration observed in SEM images is consistent with earlier findings and is typically due to van der Waals forces and insufficient stabilization.

The present SEM results are generally consistent with previous reports regarding spherical morphology and agglomeration. However, the predominance of cubic nanoparticles in leaves-derived AgNPs represents a notable variation and highlights the significant influence of plant part selection on nanoparticle morphology.

Overall, the study confirms that green synthesis conditions strongly determine nanoparticle shape and distribution, in agreement with established literature.

#### Elemental composition analysis

**Fig. 6:**
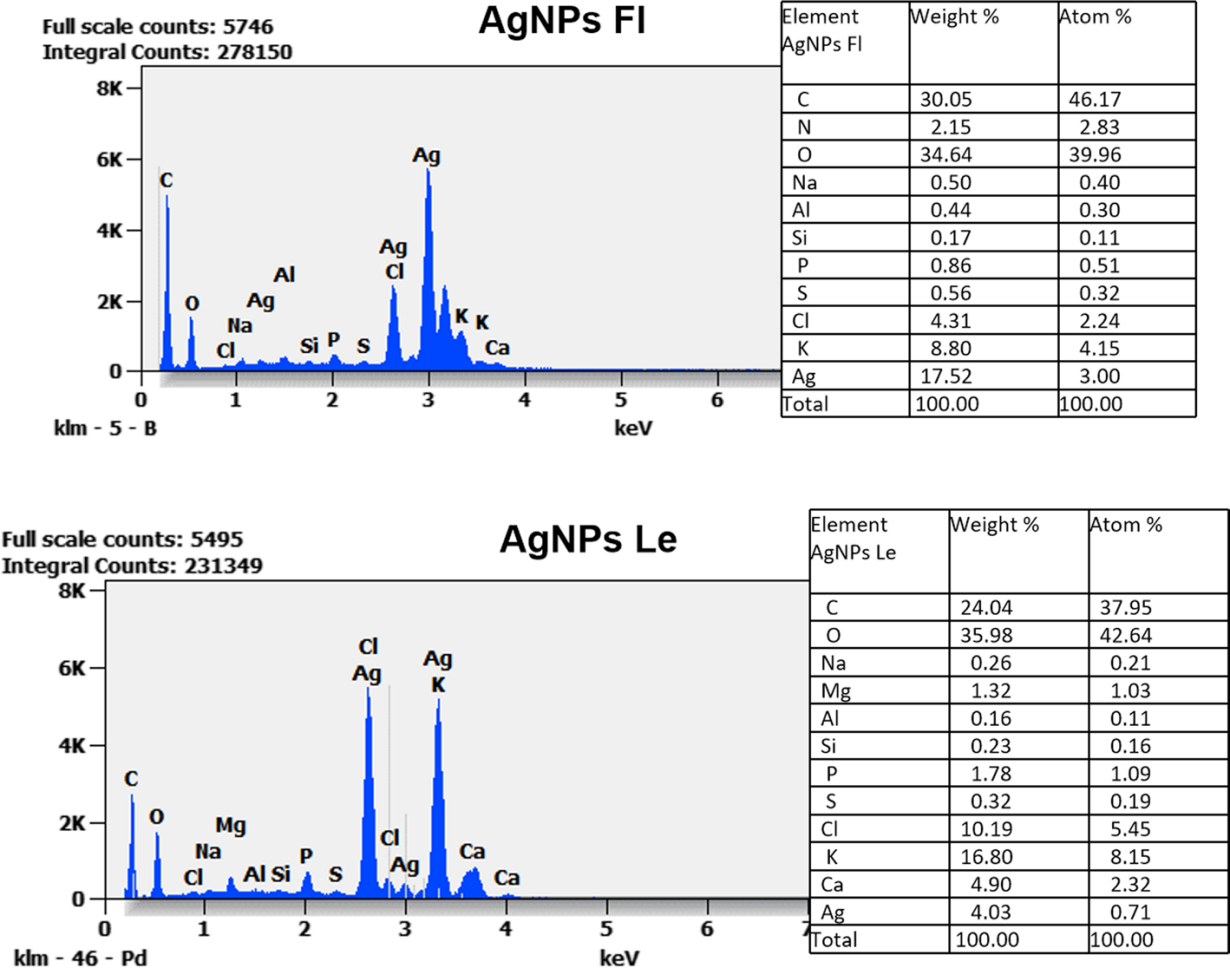
Elemental composition analysis of the two nanoparticles derived from leaves (AgNPsLe) and Flowers (AgNPsFl) of *A. arabica* determined by TES Quanta 250

Elemental analysis of both silver nanoparticles AgNPsLe and AgNPsFl measured by EDX reveals strong signals in the silver region of 3 keV and confirms the formation of silver nanoparticles with their elemental nature. Elemental analysis of silver nanoparticles revealed firstly the same elements in both nanoparticles, including carbon C, Aluminum Al, chlorine Cl, potassium (K), calcium (Ca), sulfur (S), Phosphorus (P) and Oxygen (O). These elements originate from the plant material and may be extracted during aqueous extraction. The presence of C and O acts as a natural reducing and stabilizing agents. The oxygen contributes to the surface oxidation of silver nanoparticles. S is coming from amino acids (Met, Cys) known to form strong Ag–S bonds that improve the nanoparticles stabilization (Fig. 6).

The atomic composition (atm %) reveals notable differences between the two synthesized nanoparticles. AgNPs derived from flowers (AgNPsFl) are richer in silver (3%) compared to those from leaves (AgNPsLe) at 0.71%. Regarding carbon content, AgNPsFl contain 46.14%, whereas AgNPsLe contain only 37.95%. Nitrogen (2.82%) is detected exclusively in AgNPsFl, while magnesium Mg (1.03%) is present only in AgNPsLe (Fig. 6).

Additionally, AgNPsLe derived from leaves are richer in phosphorus and sulfur than AgNPsFl. These differences in shape, size and elemental composition likely account for the variations in their biological activity, which are closely related to the nanoparticles morphology and surface characteristics.

### Biological potentialities of silver nanoparticles

#### The antioxidant activity

The DPPH assay revealed strong radical scavenging activity for the flowers (81.68%) and leaves (66.29%) extracts, which often depends more on bioactive compounds like flavonoids and polyphenols, whereas the silver nanoparticles exhibited moderate activity (< 49.37%). This may be due to the partial loss of these compounds during nanoparticles synthesis. Notably, the flowers-derived nanoparticles are spherical, which favors interaction with free radicals due to their high surface area, but still show lower DPPH activity compared to the crude extracts. In the FRAP assay, leaves-derived nanoparticles (cubic) exhibited the highest reducing power (IC_50_ = 0.139 mg/mL) compared to the flowers extract (IC_50_ = 0.765 mg/mL), suggesting that the specific faces of the nano cubes enhance electron transfer. Total antioxidant activity (TAA) ranged from 1.33–1.97, but leaves-derived nanoparticles (AgNPsLe) showed no detectable TAA, likely due to the assay’s harsh conditions (acidic pH, 90°C) (Table 1).

**Table 1:** Comparative antioxidant potential of flowers (Fl) and leaves (Le) extracts from *A. arabica* and their corresponding silver nanoparticles

	DPPH (PI%)	FRAP (IC_50_ mg/mL)	TAA mg A asc/g MS
Extract Fl	81.68 ± 2.37	0.765 ± 0.03	1.97 ± 0.13
Extract Le	66.29 ± 0.79	3.096 ± 0.18	1.63 ± 0.13
AgNPsFl	38.32 ± 3.36	5.55 ± 0.1	1.33 ± 0.92
AgNPsLe	49.37 ± 1.98	0.139 ± 0.19	*
BHT	*	0.57 ± 0.003	*
Ascorbic acid* (5mg/mL)	93.5 ± 0.02	*	*

Overall, these results indicate that the shape of the nanoparticles strongly influences their antioxidant mechanism: spherical flowers-derived AgNPsFl are more effective in radical scavenging (DPPH), whereas cubic leaves-derived (AgNPs e) excel in reducing power (FRAP) but may be less stable under certain conditions, affecting total antioxidant activity. Several works highlight the influence of the nanoparticles shape on their antioxidant activities determined by DPPH and FRAP assays [10, 77].

#### In vitro anti-inflammatory activity

**Fig. 7:**
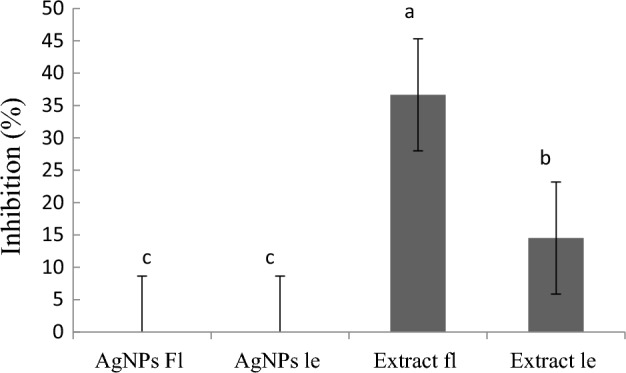
The anti-inflammatory activity of silver nanoparticles biosynthesized from flowers (AgNPsFl) and leaves (AgNPsLe) extracts were compared to that of the corresponding aqueous extracts (extract Fl and extract Le). Values followed by different letters indicate significant difference at *p* < 0.05

Inflammation in the body is associated with protein denaturation, and certain drugs can prevent or reduce this process. If a tested compound inhibits the denaturation of bovine serum albumin (BSA), it is considered to have anti-inflammatory potential. In the in vitro BSA assay, both extracts exhibited moderate anti-inflammatory activity, with inhibition percentages of 36.65 and 14.53% for the flowers and leaves extracts, respectively. In contrast, the corresponding silver nanoparticles (AgNPs Fl and AgNPs Le) showed no inhibitory effect on BSA denaturation (Fig. 7).

The phytochemical analysis showed that *A. arabica* is rich in tannins, flavonoids and polyphenols, contributing to its notable antioxidant as evidenced by assays such as DPPH and FRAP and anti-inflammatory properties [15].

#### In vitro antidiabetic capacity by α-amylase and α-glucosidase inhibition

Diabetes is recognized as one of the major global health challenges of the 21^st^ century. An effective strategy for managing type 2 Diabetes Mellitus (T2DM) involves delaying glucose absorption by regulating carbohydrate digestibility through the inhibition of starch hydrolyzing enzymes such as alpha-amylase and alpha-glucosidase thereby controlling postprandial hyperglycemia [45]

Although clinical inhibitors such as acarbose, miglitol and voglibose are commonly prescribed, their therapeutic efficacy is often constrained by undesirable side effects [14]. In this context, the present study investigates the potential of newly synthesized silver nanoparticles from leaves and flowers extracts of *A. arabica* as natural alternative enzyme inhibitors for the management of diabetes.

**Fig. 8:**
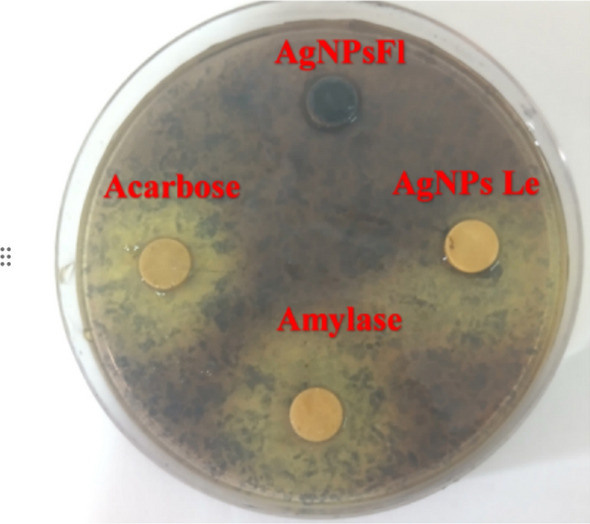
Starch agar plate assay showing inhibition of α-amylase

The results obtained from the starch agar plate clearly demonstrate the high effectiveness of AgNPsFl, which achieved complete inhibition of α-amylase activity (Fig. 8), in contrast to the weaker inhibition observed with acarbose or AgNPsLe (Table 2).

**Table 2:** Α-amylase inhibition assay on starch agar plate, (Mean ± SD)

Enzyme inhibition	Diameter zone (mm)	Inhibition activity (%)
Enzyme (alone)	30 ± 0	0 ± 0
Acarbose (40 µg)	26 ± 1.41	13.33 ± 0
AgNPsFl (30 µg)	0 ± 0	100 ± 0
AgNPsLe (40 µg)	24.5 ± 0.7	18.33 ± 0

**Fig. 9:**
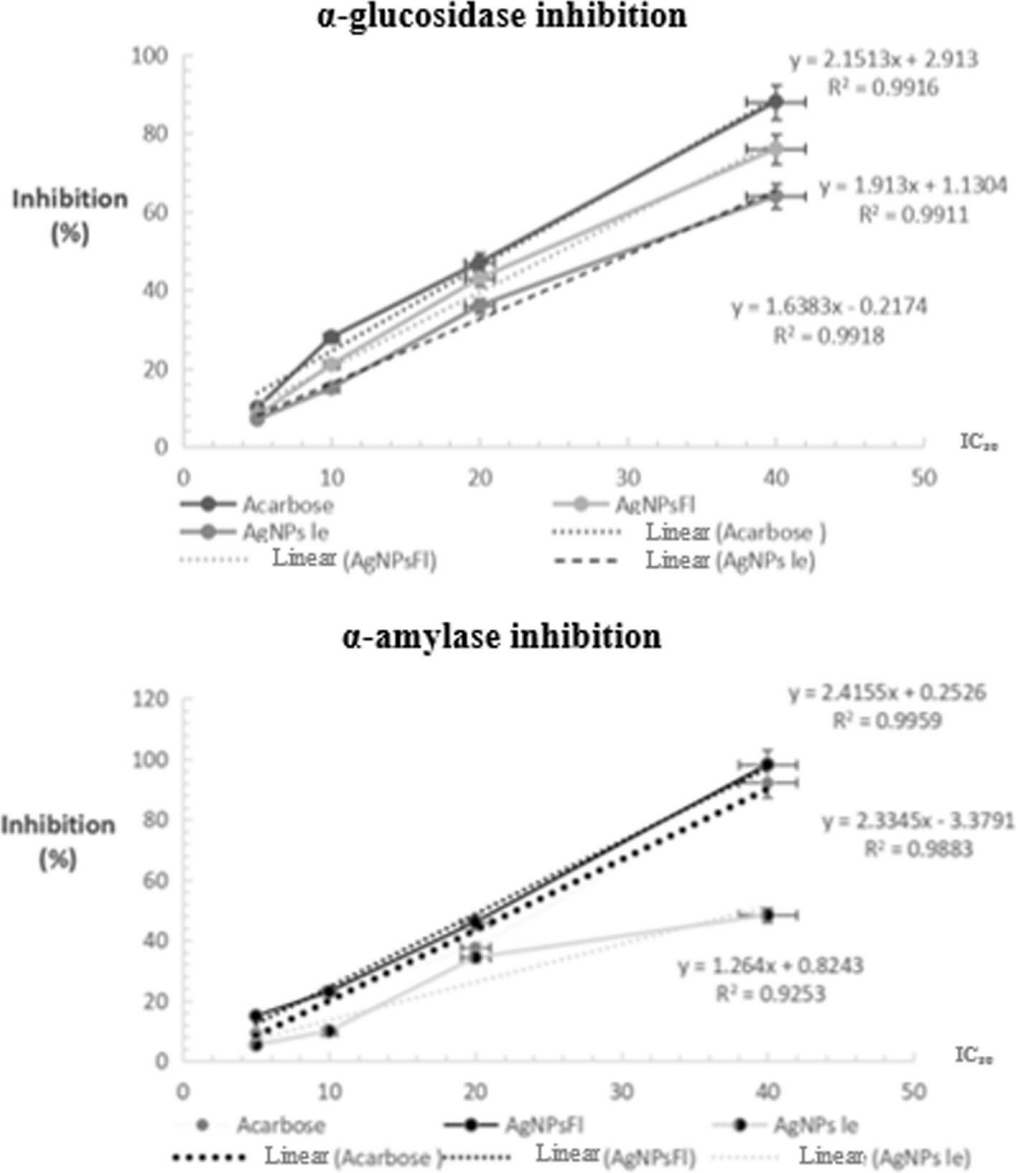
Dose-dependent inhibition of α-amylase and α-glucosidase by both described silver nanoparticles derived from *A. arabica* flowers AgNPsFl and leaves AgNPsLe compared to the commonly used standard inhibitor, Acarbose

Both AgNPsFl and AgNPsLe inhibited dose-dependent inhibition of α-amylase and α-glucosidase. The highest α-amylase inhibition was observed at 40 µg mL^− 1^, with Acarbose reaching 92.1% and AgNPsFl 89%. Several studies have highlighted the inhibitory potential of nanoparticles against α-amylase and α-glucosidase (Fig. 9).

For α-glucosidase, maximum inhibition was also observed with Acarbose at 40 µg mL^− 1^, followed by AgNPsFl at the same concentration (Fig. 9).

Since different studies use various concentrations, solvents and experimental conditions, IC_50_ values provide a more meaningful comparison than absolute inhibition percentages. Both biosynthesized nanoparticles inhibited the enzymes with IC_50_ values comparable to those reported in the literature (Table 3).

**Table 3:** IC_50_ values (Mean ± SD) for α-amylase and α-glucosidase inhibition by the biosynthesized nanoparticles and acarbose used in the current study, compared with previously reported plant extracts and nanoparticles

Samples^ref^	α-amylase inhibition (IC_50_; µg mL^− 1^)	α-glucosidase inhibition ( IC_50_; µg mL^− 1^)
Acarbose^This work^	22.87 ± 0.035	22 ± 0.544
AgNPsFl^This work^	20.59 ± 0.12	29.34 ± 1.152
AgNPsLe^This work^	31.27 ± 1.25	30.61 ± 0.975
D. reticulata extract ^Ref 1^ Acarbose^Ref 1^	– –	0.918 1.37
AgNPs^Ref 2^	16.65	18.97
Acarbose^Ref 3^	1.38	1.35
SAgNPs^Ref 3^	4.92	0.68
Extract *Achillea tenorii*^*Ref* 4^		32
Acarbose ^Ref 5^	30	40
AgNPsDL^Ref 5^	120	145

Ref 1: [43],Ref 2: [47],Ref 3: [27],Ref 4: [75],Ref 5: [52]

For α-amylase IC_50_ values ranged from 20.59 µg mL^− 1^ for AgNPsFl to 31.27 µg mL^− 1^ for AgNPsLe. For alpha-glucosidase, IC_50_ values were 22 µg mL^− 1^ for acarbose, 26.72 µg mL^− 1^ AgNPsFl and 30.65 µg mL^− 1^ for AgNPsLe. It is well known that plant extract and nanoparticles are rich in biomolecules capable of inhibiting enzymes [43, 47].

#### Antimicrobial action

The antibacterial and antifungal activities assessed by the agar well diffusion method showed that both biosynthesized silver nanoparticles exhibited strong antibacterial effects against all tested gram-positive and gram-negative bacterial strains, whereas the flowers and leaves extracts (EFl, Ele) showed no antibacterial activity under the same conditions. No antifungal activity was observed for any of the tested samples against the two Candida species. Overall, the inhibition zones produced by silver nanoparticles from the flowers extract (AgNPsFl) were larger than those from the leaves extract (AgNPsLe) (Table 4).

**Table 4:** Comparative diameter zone inhibition of *A. arabica* extracts from leaves and flowers and their derived silver nanoparticles

Microorganisms strains	Diameter of zone inhibition (mm) (Mean ± SD)				
	AgNPs Fl	AgNPsLe	Flowers extract Fl	Leaves extract Le	Standards
*Bacterial species*					Levofloxacin 15 µg mL^− 1^
*E.coli* ATCC 35218	15^c^ ± 0.01	12^d^ ± 0.0	0 ± 0.0	0 ± 0.0	15^e^ ± 0.0
*Listeria monocytogenes* ATCC 19115	15^c^ ± 0.0	12^d^ ± 0.032	0 ± 0.0	0 ± 0.0	32^a^ ± 0.01
*Staphylococcus aureus* ATCC 43300	17^b^ ± 0.01	12^d^ ± 0.0	0 ± 0.0	0 ± 0.0	12^f^ ± 0.05
*Staphylococcus epidermidis* CIP 106510	14.5^c^ ± 0.015	14^c^ ± 0.01	0 ± 0.0	0 ± 0.0	30^b^ ± 0.02
*Salmonella* Typhimurium ATCC 14028	17^b^ ± 0.032	15^b^ ± 0.0	0 ± 0.0	0 ± 0.0	32^a^ ± 0.005
*Bacillus cereus* ATCC 14579	28^a^ ± 0.005	20^a^ ± 0.15	0 ± 0.0	0 ± 0.0	32^a^ ± 0.0
*Enterococcus faecalis* (ATCC 29212)	14^d^ ± 0.0	14^c^ ± 0.05	0 ± 0.0	0 ± 0.0	25^c^ ± 0.015
*Fungal species*					Amphotericin B(30 µg mL^− 1^)
*Candida albicans*	0^e^ ± 0.0	0^e^ ± 0.01	0 ± 0.015	0 ± 0.01	18^d^ ± 0.01
*Candida krusei*	0^e^ ± 0.0	0^e^ ± 0.0	0 ± 0.0	0 ± 0.0	15^e^ ± 0.005

Values followed by different letters in the same columns indicate the significant difference at *p* < 0.05.

The MIC and MBC results confirmed the potent antibacterial activity of flower-derived silver nanoparticles (AgNPsFl), with MIC values ranging from 37.5–75 µg mL^− 1^, compared to 75 to 150 µg mL^− 1^ for leaves-derived nanoparticles (AgNPsLe). The MBC/MIC ratios further indicate that both types of nanoparticles exhibit a bactericidal effect (Table 5).

**Table 5:** Determinations of MIC and MBC values of AgNPs Fl and AgNPs Le against the tested pathogens

Microorganisms strains	AgNPsFl (Mean ± SD)			AgNPsLe (Mean ± SD)		
Bacterial strains	MIC	MBC	Ratio MBC/MIC	MIC	MBC	Ratio MBC/MIC
*E.coli* ATCC 35218	75^c^ ± 0.15	150^b^ ± 0.01	2	150^b^ ± 0.015	300^a^ ± 0.0	2
*Listeria monocytogenes* ATCC 19115	75^c^ ± 0.005	150^b^ ± 0.032	4	150^b^ ± 0.01	300^a^ ± 0.01	2
*S.aureus* ATCC 43300	37.5^d^ ± 0.01	75^c^ ± 0.01	2	75^c^ ± 0.05	300^a^ ± 0.01	4
*S.epidermidis* CIP 106510	37.5^d^ ± 0.01	150^b^ ± 0.0	4	75^c^ ± 0.032	300^a^ ± 0.01	4
*Salmonella* Typhimurium ATCC 14028	75^c^ ± 0.017	150^b^ ± 0.01	4	150^b^ ± 0.015	300^a^ ± 0.0	2
*Bacillus cereus* ATCC 14579	37.5^d^ ± 0.0	150^b^ ± 0.01	4	75^c^ ± 0.01	300^a^ ± 0.017	4
*Enterococcus faecalis* (ATCC 29212)	75^c^ ± 0.005	150^b^ ± 0.0	4	150^b^ ± 0.0	300^a^ ± 0.05	2

Values are expressed in µg mL^− 1^. The ratio MBC/MIC < 4

Values followed by different letters are significantly different at *p* < 0.05

The stronger antibacterial action observed for spherical silver nanoparticles (AgNPsFl) compared to cubic shaped leaves-derived nanoparticles (AgNPsLe) can be explained by differences in shape and size. Spherical nanoparticles generally have a higher surface area to volume ratio, which enhances their interaction with bacterial cell membranes and facilitates greater adsorption of silver ions [24]. Additionally, spherical nanoparticles are more readily internalized by bacterial cells, allowing more effective delivery of silver ions directly into the cytoplasm [34]. Their smaller size of about 16 nm and spherical shape also promote faster silver ion release, further increasing their antibacterial efficacy [49]. These factors collectively explain why AgNPs Fl exhibited larger inhibition zones against both gram-positive and gram-negative bacteria compared to AgNPsLe (with cubic shape and smaller size of 13 nm). These findings were confirmed by the atomic composition (atm%) of AgNPs derived from flowers (AgNPsFl), which are richer in silver (3%) compared to those from leaves (AgNPsLe) at 0.71%.

Biofilm inhibition assays revealed that silver nanoparticles derived from flowers (AgNPsFl) exhibited stronger anti-biofilm activity, with inhibition percentages ranging from 44.24–64.38%, compared to 32.8 to 53.14% for leaves-derived nanoparticles (AgNPsLe). The leaves-derived nanoparticles (AgNPsLe) displayed higher efficacy, especially against gram-positive biofilms, with inhibition values ranging from 43.2–53.14% which may be due to their smaller size of 13 nm (Table 6).

**Table 6:** Biofilm inhibition of tested microorganisms treated individually with the silver nanoparticles derived from flowers (AgNPsFl) and derived from leaves (AgNPsLe) of *A. arabica* tested at 150 µg mL^− 1^

Bacterial strains	Percentage of biofilm inhibition (%) (mean ± SD)	
	Flower-derived AgNPsFl	Leaf-derived AgNPsLe
*E.coli* ATCC 35218	49.1^d^ ± 0.0	38.27^f^ ± 0.01
*Listeria monocytogenes* ATCC 19115	49.7^d^ ± 0.005	36.87^f^ ± 0.005
*S.aureus* ATCC 43300	63.14^a^ ± 0.017	49.10^d^ ± 0.0
*S.epidermidis* CIP 106510	44.24^e^ ± 0.0	53.14^c^ ± 0.15
*Salmonella* Typhimurium ATCC 14028	64.38^a^ ± 0.01	32.8^f^ ± 0.05
*Bacillus cereus* ATCC 14579	58.02^b^ ± 0.0	45.16^e^ ± 0.01
*Enterococcus faecalis* (ATCC 29212)	44.24^e^ ± 0.032	43.2^e^ ± 0.0

Values followed by different letters are significantly different at *p* < 0.05

#### Antimicrobial activity and mechanism of action

The strong antibacterial activity of flowers-derived nanoparticles (AgNPsFl) against all tested bacterial strains can be attributed to their higher lysozyme like activity, particularly against *Bacillus cereus* and *Staphylococcus aureus* with values of 46.28 and 42.95 AU/mL/min, respectively, whereas leaves-derived nanoparticles (AgNPsLe) showed no detectable lysozyme activity (Table 7). This also reflects the abundance of bioactive molecules in the flowers extract capable of exhibiting lysozyme like activity, especially against Bacillus cereus. Furthermore, evaluation of exopolysaccharides (LPS) production showed that both AgNPs strongly inhibited LPS synthesis, depending on the bacterial species tested. The corresponding plant extracts also significantly affected LPS related activity, through to a lesser extent.

**Table 7:** Lysozyme Like and LPS degradation activities of silver nanoparticles (AgNPsFl) and (AgNPsLe), mean ± SD

Samples	LPS degradation (%)		Lysozyme action (AU/mL/min)	
	*E coli*	*S. typhi*	*S. aureus*	*B.cereus*
AgNPsFl	42.79 ± 0.07	61.07 ± 0.05	46.28 ± 0.032	46.95 ± 0.016
AgNPsLe	46.56 ± 0.03	58.8 ± 0.02	0 ± 0.032	0 ± 0.026

Silver nanoparticles derived from flowers (AgNPsFl) contain higher amounts of silver ions. This high Ag^+^ content, along with the positive surface charge of AgNPsFl, facilitates strong interactions with negatively charged components of bacterial cell walls, such as teichoic acids in gram-positive bacteria and lipopolysaccharides in gram-negative bacteria, leading to cell wall destabilization and increased permeability [49].

Silver nanoparticles (AgNPs) synthesized using biological extracts often carry a positive surface charge, facilitating electrostatic interactions with the negatively charged lipopolysaccharides (LPS) on bacterial cell membranes [18]. This interaction enhances the attachment of AgNPs to the bacterial surface, disrupting the integrity of the LPS layer, increasing membrane permeability, and silver by interacting with negative biomolecules such as phosphorus and sulfur, thereby causing membrane damage and morphological changes [50, 51].

The superior antibacterial efficacy of flower-derived silver nanoparticles (AgNPsFl) over leaves-derived (AgNPsLe) can be attributed to differences in their shape, silver content and surface charge. Flower-derived (AgNPsFl) are typically spherical, which provides a higher surface to volume ratio, facilitating more effective interactions with bacterial membranes. Additionally, these nanoparticles (AgNPsFl) tend to have a higher silver content, which can lead to increased antibacterial efficacy. The stronger antibacterial effect of flowers-derived nanoparticles is due to their spherical shape, higher silver ion content, and favorable surface charge, which together enhance their interaction and disruption of both gram-negative and gram-positive membranes [30].

## Discussion

### Green synthesis and characterization of *A. arabica*–mediated silver nanoparticles

This work provides the first comprehensive evidence of successful green synthesis of silver nanoparticles (AgNPs) using aqueous extracts of *A. arabica* leaves and flowers. The rapid color change from pale yellow to dark brown upon addition of AgNO_3_ is a typical indicator of nanoparticle formation and results from surface plasmon resonance (SPR), which arises from the collective oscillation of conduction electrons on the nanoparticle surface when excited by light [20, 74].

UV–Vis spectral analysis revealed characteristic SPR peaks at 450 nm for AgNPsLe and 490 nm for AgNPsFl, confirming AgNP formation. Variations in SPR peak positions are commonly associated with differences in particle size, morphology, and surface chemistry, with red shifts toward longer wavelengths indicating larger particle size or stronger interactions with phytochemical capping agents [20]. Similar spectral profiles have been reported in numerous plant‑mediated AgNP syntheses involving polyphenol‑rich extracts, wherein flavonoids and phenolics regulate nanoparticle nucleation and growth kinetics [16].

FTIR analysis further corroborated the presence of functional groups typical of plant secondary metabolites, such as hydroxyl (–OH), carbonyl (C = O), and amine (–NH) groups. These biomolecules act simultaneously as reducing agents, facilitating conversion of Ag^+^ to metallic Ag^0^, and as stabilizers preventing aggregation of nanoparticles [17, 20, 55]. Plant polyphenols are known to donate electrons during reduction while forming an organic stabilizing layer that enhances colloidal stability and biological functionality [17].

The XRD patterns revealed a crystalline structure of the synthesized AgNPs, with diffraction peaks at 2θ = 38.1°, 44.3°, 64.5°, and 77.4°, corresponding to the (111), (200), (220), and (311) planes of face‑centered cubic (fcc) silver. These match well with the standard silver reference pattern (JCPDS No. 04‑0783), confirming the formation of crystalline AgNPs [17, 20]. The average crystallite size determined via the Scherrer equation ranged from approximately 13–20 nm, which is consistent with reports for *Achillea*-derived AgNPs [13, 37].

(SEM) revealed clear morphological differences between nanoparticles synthesized from leaf and flower extracts. AgNPs synthesized from leaves (AgNPsLe) predominantly exhibited cubic structures, whereas those obtained from flowers (AgNPsFl) displayed mainly spherical morphology. Such variations in nanoparticle shape are commonly attributed to differences in the phytochemical composition of plant organs, which can influence the nucleation rate and crystal growth pathways during the reduction of silver ions [20]. Plant secondary metabolites—including flavonoids, phenolic acids, terpenoids, and proteins—can act as selective capping agents that adsorb onto specific crystallographic facets of growing nanoparticles. This selective binding modulates surface energy and growth kinetics of individual crystal planes, ultimately directing nanoparticle morphology and size distribution (Vanlalveni et al., 2021, Velgosová et al., 2024). Consequently, variations in metabolite composition between leaves and flowers may lead to different stabilization mechanisms and growth orientations, resulting in distinct nanoparticle geometries.

EDX confirmed the presence of elemental silver with characteristic signals around ~ 3 keV, a recognized fingerprint of metallic AgNPs; correlatively, AgNPsFl (~ 3% Ag) contained a higher silver signal than AgNPsLe (~ 0.7% Ag), suggesting flower extracts may harbor more potent reducing phytochemicals [17].

Collectively, these findings establish that *A. arabica* extracts serve as an effective biological platform for green AgNP synthesis, and that differential plant phytochemistry markedly influences nanoparticle morphology and physicochemical properties, consistent with previous reports in plant‑mediated nanotechnology [20]

### Correlation between phytochemical richness and antioxidant capacity

The DPPH and FRAP assays revealed strong antioxidant potential in both crude extracts and synthesized AgNPs. The flowers extract showed the highest DPPH radical scavenging (81.68%), while leaves-derived AgNPs displayed the strongest ferric-reducing power (FRAP IC_50_ = 0.139 mg/mL). The reduced radical-scavenging ability of the nanoparticles compared with crude extracts suggests partial utilization of phenolic compounds during Ag^+^ reduction; however, the retained redox activity demonstrates the persistence of surface-bound bioactive moieties.

**Fig. 10:**
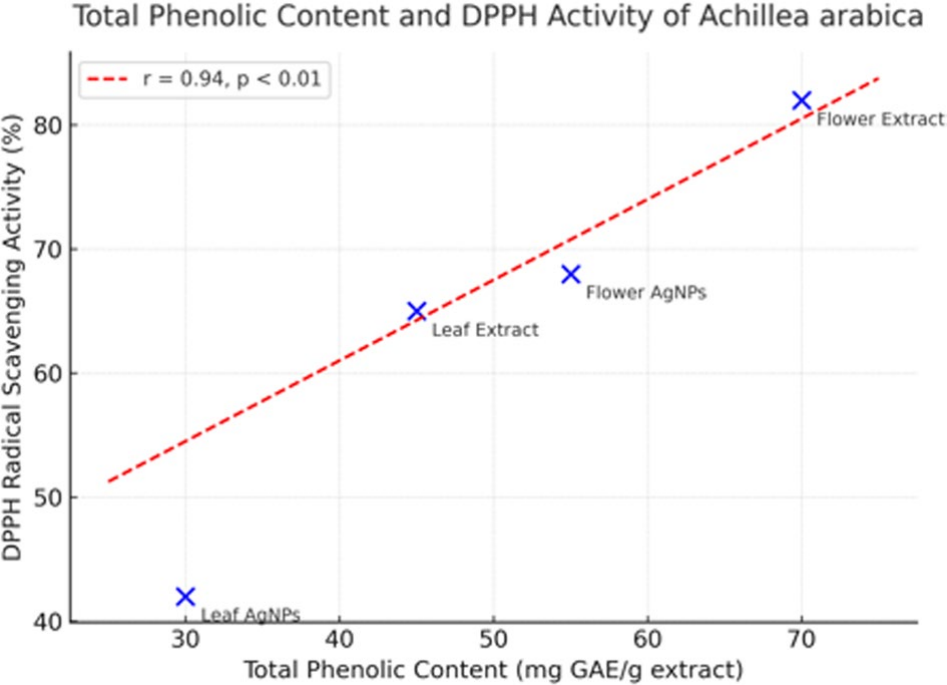
Correlation between phenolic content and antioxidant activity for *A. arabica* extracts and their AgNPs

The relationship between antioxidant activity and phytochemical content was illustrated by a positive correlation (r = 0.94) between total phenolic content and DPPH inhibition (Fig. 10). This strong linear association indicates that phenolics are the primary contributors to both n synthesis and antioxidant performance, confirming their dual functional role as reducing and stabilizing agents.Nevertheless, this correlation is based on the present sample set and assay conditions and should be interpreted within this experimental scope**.**

### Anti-inflammatory and enzyme-inhibition potential

The protein-denaturation assay demonstrated moderate anti-inflammatory effects for both extracts, especially the flowers extract, which may be attributed to its higher flavonoid and tannin levels [15]. AgNPs themselves displayed limited inhibition in the BSA denaturation assay, potentially due to altered protein–nanoparticle interactions under the test conditions. While the present findings are limited to this in vitro protein-denaturation model, the observed bioactivity may warrant further investigation in cell-based and in vivo systems to determine whether anti-inflammatory effects occur via pathways such as redox modulation and cytokine signaling [44].

The α-amylase and α-glucosidase inhibition assays revealed that AgNPsFl were potent enzyme inhibitors (IC_50_ = 20.6 and 29.3 µg mL^− 1^, respectively). To the best of our knowledge, comparable quantitative data for *A. millefolium* or A. *wilhelmsii*-derived AgNPs have not yet been reported. Previous studies on A. *millefolium*-AgNPs primarily investigated antioxidant and antibacterial properties [67], underscoring the novelty of the present antidiabetic findings for *A. arabica*. The strong inhibition observed here likely arises from synergistic interactions between the silver core and residual phytochemical ligands on the nanoparticles surface, which facilitate binding to enzyme active sites and hinder substrate access [45]. These findings suggest that *A. arabica* AgNPs may serve as bioactive nanomaterials for enzyme inhibition in vitro; however, their efficacy and safety require further validation in more complex biological systems.

### Antimicrobial and antibiofilm performance

Both types of AgNPs exhibited strong antibacterial effects, whereas the crude extracts were largely inactive, confirming that nanoparticles formation is key to biological efficacy. AgNPsFl produced larger inhibition zones and lower MIC values (37.5–75 µg mL^− 1^) than AgNPsLe (75–150 µg mL^− 1^), demonstrating a twofold improvement in antimicrobial efficiency compared to A. *wilhelmsii*-derived AgNPs reported by [13]. This between-study comparison should be interpreted cautiously because differences in synthesis conditions, bacterial strains, inoculum size, media, and assay protocols can affect MIC values and inhibition zones.

The enhanced activity of AgNPsFl can be attributed to their smaller size, spherical shape, which provides a higher surface-to-volume ratio, improved Ag^+^ ion release, and more efficient electrostatic interaction with bacterial cell walls [51]. Their biofilm inhibition (64%) was particularly notable and indicates substantial in vitro activity against biofilm formation.

Mechanistic assays demonstrated that AgNPsFl possess lysozyme-like activity and can disrupt lipopolysaccharides synthesis, confirming multi-target antibacterial actions involving oxidative stress generation, membrane disruption, and interference with polysaccharides metabolism. Such multifaceted antibacterial behavior supports the candidacy of *A. arabica*-derived AgNPs for further investigation as alternative antimicrobial materials or surface coatings, pending biocompatibility and efficacy validation in more complex bioassays.

### Structure–function relationships and mechanistic insight

Comparative analysis showed that particle geometry, silver content, and surface chemistry directly influence biological outcomes. The spherical AgNPsFl excelled in antimicrobial and enzyme-inhibition assays due to their greater reactive surface area and electron density, while cubic AgNPsLe exhibited superior reducing antioxidant power due to efficient electron-transfer through crystallographic faces.

This correlation highlights a key structure–function relationship: morphology dictates biological interaction efficiency. Smaller, spherical particles penetrate microbial membranes and enzyme active sites more effectively, whereas larger cubic particles provide enhanced redox stability. These biological interpretations are based on the observed physicochemical features and in vitro outcomes and should be further validated using targeted molecular and cellular assays.Such relationships provide a rational framework for tailoring nanoparticles design according to therapeutic needs.

### Translational and pharmaceutical applications

Given their potent antimicrobial, antioxidant, and antidiabetic activities, *A. arabica*-derived AgNPs are excellent candidates for translational research. These nanoparticles can be integrated into AgNP-loaded wound dressings, biopolymeric nanogels for diabetic ulcers, or antioxidant nanocreams for skin infections, offering multi-mechanistic protection through microbial inhibition, oxidative stress reduction, and metabolic regulation. However, such applications remain prospective at this stage and require additional evidence on cytocompatibility, formulation stability, dose optimization, and in vivo efficacy.

Additionally, the use of aqueous extracts ensures safety, cost-effectiveness, and environmental compatibility, aligning with green chemistry and sustainable nanomedicine principles. Scaling up such biosynthesis can provide pharmaceutical-grade nanomaterials without the need for hazardous chemicals or complex instrumentation. Nonetheless, scale-up and batch-to-batch reproducibility should be systematically assessed to ensure consistent physicochemical properties and bioactivity.

### Comparative novelty and scientific significance

Compared with previously published work on other *Achillea* species, *A. arabica*-derived AgNPs show favorable physicochemical and strong in vitro bioactivity. For instance, AgNPs from *A. millefolium* and *A. wilhelmsii* were predominantly spherical (20–40 nm) with moderate antibacterial and antioxidant activities [13, 37]. In contrast, the present work demonstrated dual morphology (spherical and cubic), smaller particle sizes (13–20 nm), and significantly lower MIC and IC_50_ values, reflecting enhanced bioactivity These comparisons should be interpreted with caution, as differences in experimental design and assay conditions across studies can influence the reported results…

This dual-organ approach (leaves vs. flowers) further represents a methodological advancement, revealing how organ-specific phytochemical variation modulates nanoparticles structure and function. Such comparative insight enhances both the originality and mechanistic understanding of green nanofabrication using medicinal plants.

Table 8 presents a comparative overview of the biological activities of silver nanoparticles synthesized from *A. arabica* leaves and flower-derived nanoparticles with those reported in previous studies. The results indicate that AgNPsFl exhibit stronger antidiabetic and antibacterial activities compared with many previously reported plant mediated silver nanoparticles (Table 8).

**Table 8:** Comparative table of the biological potential of the silver nanoparticles derived from *A. arabica* leaves and flowers and those reported in previous studies

AgNPs (sources)	α-amylase inhibition IC_50_ (µg mL^− 1^)	α-glucosidase inhibition IC_50_ (µg mL^− 1^)	Antioxidant activity DPPH or FRAP	Antibacterial activity	Reference
AgNPs Fl (*Achillea arabica* flowers	20.59	29.34	Moderate activity FRAP IC_50_ = 765 µg mL^− 1^	MIC: 37.5–75 µg mL^− 1^	This work
AgNPs Le (*Achillea arabica* leaves)	31.27	30.61	Moderate activity FRAP IC_50_ = 139 µg mL^− 1^	MIC: 75–150 µg mL^− 1^	This work
AgNPs (*Achillea maritima*)	64.9	41.6	DPPH IC_50_ 89.46	Significant antimicrobial activity	[20]
AgNPs (*Rosa indica* petals)	50–75	50–75	Strong antioxidant activity	Large spectrum of activity	[7]
AgNPs (*Brachychiton populneus* leaves)	67	–	DPPH IC_50_ = 33.85 µg mL^− 1^	–	[56]
AgNPs from *D. reticulata*	–	0.918	High antioxidant activity	Antibacterial activity	Kumkarai et al. (2015)
AgNPs Plant extract	4.92	0.68	High antioxidant potential	Broad antibacterial spectrum	[28]
AgNPs *F.cretica*	118	250	DPPH IC_50_ = 210 µg mL^− 1^ ABTS IC_50_ = 37 µg mL^− 1^	–	[41]
AgNPs DL (*D.longifolia*)	120	145	DPPH IC_50_ = 80 µg mL^− 1^	Antibacterial activity (ZI30-40mm)	[52]

The comparative analysis indicates that the biosynthesized silver nanoparticles from *A. arabica* flowers AgNPsFl exhibit strong inhibitory activity against both α-amylase and α-glucosidase enzymes, with IC_50_ values comparable to or lower than several previously reported plant mediated AgNPs (Table 8). Moreover, the antibacterial activity of AgNPs Fl with MIC values ranging from 37–75µg, demonstrates a potent bactericidal effect against botrh gram positive and gram negative bacteria. These finding highlight the promising biomedical potential of *A. arabica* derived nanoparticles as natural alternatives for antimicrobial antidiabetic applications.

## Conclusion

This study provides the first comprehensive demonstration of the green synthesis of silver nanoparticles (AgNPs) using aqueous extracts of *A. arabica* leaves and flowers. The biogenic synthesis route proved to be an eco-friendly, reproducible, and sustainable method for producing crystalline nanosilver with distinct physicochemical and biological characteristics. Morphological and elemental analyses confirmed that differences in atomic composition, particle size, and geometry particularly the spherical shape of flower-derived AgNPs versus the cubic morphology of leaf-derived AgNPs played a decisive role in defining their biological activity profiles.

Both nanoparticles types exhibited significant antioxidant, antidiabetic, and antimicrobial effects, supporting their multifunctional bioactivity under the in vitro conditions investigated.. The spherical flower-derived AgNPs demonstrated stronger in vitro biological activity than leaf-derived AgNPs, and this enhancement is plausibly related to their greater silver content, enhanced surface-to-volume ratio, and stronger physicochemical interactions with biomolecules and microbial membranes These features collectively promoted more efficient cellular integration, redox modulation, and enzymatic inhibition compared with cubic leaf-derived counterparts.

Overall, *A. arabica* acts as a powerful biogenic platform for the synthesis of multifunctional AgNPs and as a renewable, high-value resource for sustainable nanopharmaceutical development. However, the present findings are limited to physicochemical characterization and in vitro assays, and they do not by themselves establish in vivo efficacy or safety**.** Integrating its phytochemical matrix into nanosilver systems offers a rational basis for further exploration of therapeutic materials targeting oxidative stress, metabolic dysregulation, and microbial infections. Future research should focus on in vivo pharmacological and toxicological evaluations, biocompatibility assessment, and formulation of *A. arabica*-based AgNPs into targeted delivery systems such as hydrogels or wound-healing nanogels to further substantiate safety and efficacy prior to clinical application.

## Data Availability

All data generated or analyzed during this study are included in this published article [and its supplementary information files].

## Declarations

### Clinical trial number

Not applicable. This study is not a clinical trial and does not involve any clinical interventions.
